# From chemical signatures to consumer preference: Decoding bottle and barrel aging effects on *Aronia melanocarpa* wines using integrated analytical and sensory techniques

**DOI:** 10.1016/j.fochx.2026.103537

**Published:** 2026-01-13

**Authors:** Zhongzheng Zhang, Qichen Yuan, Hailong Xu, Mingbo Li, Xu Zhao

**Affiliations:** a School of Life Sciences, Yantai University, Yantai, Shandong 264006, China; b Institute of Blue Economic Research, Weihai, Shandong 264300, China

**Keywords:** *Aronia melanocarpa*, Wine, Aging, HS-GC-IMS, CATA

## Abstract

This study comprehensively evaluated the phytochemical and organoleptic evolution of *Aronia melanocarpa* wines aged for 12 months in glass bottles and oak barrels. Integrated analytical approaches—including CIELab color space, phenolic profiling, antioxidant capacity assays, headspace-gas chromatography-ion mobility spectrometry (HS-GC-IMS), electronic tongue, and Check-All-That-Apply (CATA) consumer testing—revealed distinct aging dynamics. Barrel aging caused 96.2% anthocyanin loss, shifting wine color to reddish-brown, while bottle aging preserved greater color stability (retaining 42.6% of anthocyanins). HS-GC-IMS identified 65 volatile compounds; barrel aging increased oxidative markers (e.g., 3-penten-2-one and 4-hydroxy-4-methyl-2-pentanone) and oak-derived notes (e.g., cocoa and coconut), whereas bottle aging enhanced tertiary aromas characterized by spicy, tobacco, and leather nuances. Electronic tongue and CATA analyses confirmed both aging methods improved taste profiles by reducing sourness and mitigating astringency/bitterness aftertastes. Bottle-aged wine demonstrated superior consumer preference (54.24% first-choice ranking) due to its fruity aroma, deeper color, and balanced taste.

## Highlights

• First systematic study on bottle vs. oak‑barrel aging effects on Aronia melanocarpa wine chemistry and sensory properties.
• Barrel aging caused 96.2% anthocyanin loss; bottle aging retained 42.6%, ensuring better color stability.
• HS-GC-IMS identified 65 volatiles: barrels raised oxidative/oak notes; bottles enhanced tobacco, leather aromas.
• Electronic tongue and CATA confirmed both aging methods improved taste by reducing sourness and astringency/bitterness.
• Bottle-aged wine was significantly preferred by consumers (54.24% first choice) due to fruity aroma and balanced taste.

## 1 Introduction

*Aronia melanocarpa* (black chokeberry), a Rosaceae shrub indigenous to North America and now cultivated worldwide, has recently expanded into China's eastern provinces (Borowska & Brzóska, 2016; Shi, Xu, Sheng, & Song, 2024). The berries are highly valued for their exceptional concentration of bioactive phenolics, particularly anthocyanins and proanthocyanidins, which contribute to significant health-promoting benefits (Dobros, Zielińska, Siudem, Zawada, & Paradowska, 2024). However, the pronounced astringency of fresh fruit limits direct consumption (Huang, Fang, Xie, Liu, & Xu, 2022; Kang, Kang, Lee, & Chang, 2018), driving the development of value-added products such as juices, jams, and dietary supplements (Shi et al., 2024). Winemaking offers an effective utilization pathway for *Aronia melanocarpa*, capitalizing on its high juice yield and bioactive phenolic profile to create distinctive wines with extended shelf-life, enhanced market value, and diversified commercial potential.

In our preceding work (Yuan et al., 2025), we have systematically elucidated the influence of key vinification parameters—specifically maceration duration and malolactic fermentation on the chromatic attributes, phenolic content, aroma composition, and sensory perception of *Aronia melanocarpa* wines. While this study established a foundational framework for the targeted production of *Aronia melanocarpa*-based wines, the critical role of aging remains unexplored. Aging is widely acknowledged as a prerequisite for the production of premium wines, where controlled oxidation, esterification, and polymerization reactions progressively transform harsh young wines into stable and complex wines characterized by softened tannins, mellowed acidity, and a refined bouquet (Echave, Barral, Fraga-Corral, Prieto, & Simal-Gandara, 2021; Zhang et al., 2020). Traditionally, oak barrels facilitate micro‑oxygenation and contribute oak-derived volatile compounds (e.g., oak lactones, vanillin, and guaiacol), thereby enhancing structural integrity and conferring characteristic coconut, wood, and smoky notes (Carpena, Pereira, Prieto, & Simal-Gandara, 2020; Ma et al., 2022). Conversely, bottle aging relies on minimal oxygen ingress—from residual bottling oxygen or closure permeation—to drive slower oxidative and hydrolytic reactions, refining aroma and palate without oak-derived notes (Vázquez-Pateiro, Arias-González, Mirás-Avalos, & Falqué, 2020; Zhang et al., 2023). Although these mechanisms are well-established for *Vitis vinifera* wines, their effects on *Aronia melanocarpa* wines remain unknown.

Therefore, the present study provided the first systematic evaluation of glass bottle and oak barrel aging on the chemical and sensory evolution of *Aronia melanocarpa* wines over 12 months. By integrating CIELab color space methodology, targeted phenolic analyses, antioxidant capacity assays, headspace-gas chromatography-ion mobility spectrometry (HS-GC-IMS), electronic tongue, and Check-All-That-Apply (CATA) consumer testing, the findings could offer valuable guidance for developing applicable aging protocols to enhance the organoleptic quality and market acceptance of *Aronia melanocarpa* wines.

## 2 Materials and methods

### 2.1 Reagents and standards

Analytical grade potassium chloride, sodium chloride, sodium acetate, sodium hydroxide, ammonium sulfate, and methyl cellulose (viscosity = 1500 mPa·s) were purchased from Beijing Solarbio Science & Technology Co., Ltd. (Beijing, China). Analytical grade 1,1-diphenyl-2-picrylhydrazyl (DPPH), 2,2′-azino-bis-3-ethylbenzthiazoline-6-sulphonic acid (ABTS), 6-hydroxy-2,5,7,8-tetramethylchroman-2-carboxylic acid (Trolox), and hydrochloric acid were purchased from Sinopharm Chemical Reagent Co., Ltd. (Shanghai, China). Chromatographic grade ethanol, methanol, and dichloromethane were obtained from Honeywell (Marris, Township, NJ, USA). Phenolic standard of (−)-epicatechin (CAS: 490–46-0) with a purity of ≥98% was purchased from Beijing Solarbio Science & Technology Co., Ltd. (Beijing, China). The standards of volatile compounds used for quantification were purchased from Sigma-Aldrich (St. Louis, MO, USA) (Table S1). Deionized water (resistivity = 18.2 MΩ‧cm at 25 °C) was produced using a Milli-Q Element water purification system (Millipore, Bedford, MA, USA).

### 2.2 Conduct of vinification

*Aronia melanocarpa* ‘Fukangyuan 1’ berries were harvested at commercial maturity (14°Brix) from Weihai, Shandong, China, in 2023. The vinification process was carried out according to our previous method (Yuan et al., 2025). Briefly, hand-crushed berries were transferred to 20-L stainless steel fermentation tanks. Potassium metabisulfite (88 ppm) and pectinase (1.5 g/hL) (Laffort, Bordeaux, France) were added immediately. Activated yeast (F33, 20 g/hL; Laffort, Bordeau, France) was inoculated, followed by sugar supplementation to 220 g/L initial concentration. Alcoholic fermentation (18–20 °C) included twice-daily punch-downs. Pomace was removed and pressed after 72 h. After alcoholic fermentation, the wine was racked into new tanks, inoculated with lactic acid bacteria (B7 Direct, 1 g/hL; Laffort, Bordeau, France), and underwent malolactic fermentation (16–18 °C). Fermentation completion was verified by paper chromatography.

Upon completion of malolactic fermentation, wines were racked and supplemented with potassium metabisulfite (88 ppm). At this point, the basic physicochemical parameters of wine were as follows: ethanol 12.5% (*v*/v), pH 4.20, total acid 6.8 g/L (expressed as tartaric acid equivalents), residual sugar 1.8 g/L, free SO₂ 18 mg/L, and total SO₂ 95 mg/L. They were then allocated to two types of aging vessels: three new, first-use 8-L French oak barrels (*Quercus petraea* Liebl, medium-toasted and fine grain) and twelve 750-mL dark brown glass bottles (sealed with natural corks). Minimal headspace was maintained in all vessels after filling. All vessels underwent aging in temperature-controlled cabinets (16 ± 1 °C).

Samples from all aging vessels (barrels and bottles) and at each scheduled time point (e.g., 0, 3, 6, 9, 12 months) were collected, immediately frozen at −40 °C, and stored until all samples for the entire 12-month aging trial had been collected. All frozen samples were thawed uniformly under controlled conditions: at 4 °C in the dark for 12 h prior to analysis, followed by gentle homogenization. They were then analyzed together in a single, randomized batch.

### 2.3 Color measurement

Wine filtrates (0.45 μm PTFE membrane) were analyzed in 2-mm glass cuvettes using an UV–visible spectrophotometer (X-8, Metash Instruments Co., Ltd., Shanghai, China). Absorption spectra (ranging from 400 to 700 nm, 1-nm intervals) were recorded against deionized water blanks. CIELab color parameters (*L*^⁎^: lightness; *a*^⁎^: green-red axis; *b*^⁎^: blue-yellow axis; *C*^⁎^: chroma) were derived from spectral data following the standard method (10° observer, D65 illuminant) (Gordillo, Rodríguez-Pulido, Escudero-Gilete, González-Miret, & Heredia, 2012).

### 2.4 Total anthocyanin and tannin content measurement

Total anthocyanins were quantified via the pH-differential method (Wang et al., 2016). Samples were diluted in pH 1.0 (HCl/KCl) and pH 4.5 (CH₃COONa/CH₃COOH) buffers. Following 30-min equilibration, absorption spectra (ranging from 400 to 700 nm) were recorded. Concentrations were calculated as cyanidin-3-*O*-glucoside equivalents (mg/L) using Eq. (1):

(1) $$\text{Anthocyanin content}\;\left(\mathrm{mg}/L\right)=\frac{\left(\Delta \;A\times \mathrm{Mw}\times \mathrm{DF}\times 1000\right)}{\varepsilon \times l}$$

where Δ *A* = (*A*_max_-*A*_700 nm_)_pH_ _1.0_ – (*A*_max_-*A*_700 nm_)_pH_ _4.5_, Mw (molecular weight) = 449.2 g/mol, Dilution factor (DF) = 10, *ε* (molar extinction coefficient) = 26,900 L/mol/cm, and *l* refers to optical pathlength (0.2 cm in this study).

Total tannin content was determined using a modified methylcellulose precipitation method (Mercurio, Dambergs, Herderich, & Smith, 2007). Briefly, clarified wine (0.25 mL) was combined with 3 mL methylcellulose solution (0.04% *w*/*v*), incubated for 3 min, then treated with 2 mL saturated ammonium sulfate and 4.75 mL deionized water. After vortexing (10 min) and centrifugation (10,000 ×*g*, 20 min), supernatant absorbance was measured at 280 nm. Tannin content was calculated from differential absorbance (methylcellulose-treated vs. control) and expressed as (−)-epicatechin equivalents.

### 2.5 In vitro antioxidant measurement

Antioxidant capacity was evaluated using three methods: ferric reducing antioxidant power (FRAP), DPPH and ABTS radical scavenging assays. The FRAP assay was performed using a commercial BC1310 kit (Beijing Solarbio Science & Technology Co. Ltd., China) following the manufacturer's instructions. Results were expressed as Fe^2+^-TPTZ equivalents (μmoL/mL). The DPPH and ABTS assays were performed according to previously described method (Liang, Zhang, Ma, Zeng, & Fang, 2024) with slight modifications.

Briefly, for the DPPH assay, 0.1 mL of wine sample was mixed with 0.1 mL of 0.2 mM DPPH solution (sample). Control and blank reactions contained 0.1 mL wine +0.1 mL ethanol, and 0.1 mL ethanol +0.1 mL DPPH solution, respectively. After 30 min of dark incubation, absorbance was measured at 517 nm. For the ABTS assay, 20 μL of wine sample was mixed with 200 μL of ABTS working solution (sample). Control and blank reactions contained 20 μL wine +200 μL distilled water, and 20 μL distilled water +200 μL ABTS working solution, respectively. After 12 h of reaction (pre-diluted to A_734_ = 0.70 ± 0.02), mixtures were incubated in the dark for 20 min, and absorbance was measured at 734 nm. DPPH/ABTS radical scavenging activity (%) was calculated as follows:

(2) $$\mathrm{DPPH}/{\mathrm{ABTS}}^{+}\;\mathrm{radical scavening activity}\;\left(\%\right)=\left(1-\frac{\left(A_{s}-A_{c}\right)}{A_{b}}\right)\times 100\%$$

where *A*_s_ is absorbance of sample, *A*_c_ is absorbance of control, *A*_b_ is absorbance of blank. Results for both assays were expressed as Trolox equivalents (μmoL/mL).

### 2.6 Volatile compounds analysis by HS-GC-IMS

Volatile compounds in *Aronia melanocarpa* wines were detected and analyzed using an HS-GC-IMS (FlavourSpec®, G. A. S., Dortmund, Germany) following a previously described method (Wang et al., 2025) with slight modifications. First, wine samples were diluted five-fold with distilled water. Then, 2.0 mL of the diluted sample was transferred into a 20 mL headspace vial and incubated at 40 °C with stirring speed of 500 rpm for 20 min. Following incubation, 500 μL of the headspace gas was injected into the inlet through a heated syringe at 45 °C. The GC separation was performed on an MXT-WAX capillary column (15 m × 0.53 mm, 1 μm; Restek, USA) at 80 °C for 30 min, with high-purity nitrogen (99.999% purity) serving as the carrier and drift gas. The setting of the flow rate procedure was as follows: the initial flow rate of 2.0 mL/min was maintained for 2 min, rising to 15 mL/min within 8 min, then rising to 50 mL/min over the next 5 min, followed by a further rise to 100 mL/min within 5 min, and maintaining 100 mL/min for 10 min.

The identification of volatile compounds was conducted using VOCal 0.4.03 software (G.A.S., Dortmund, Germany), based on the dual match of drift time (DT) and retention index (RI) with the instrument's built-in library. The NIST 2020 database (https://webbook.nist.gov/chemistry/name-ser/) was used for supplementary RI comparison. Notably, identifications were not confirmed with authentic standards in this study, as the primary objective was to profile relative changes during aging rather than to achieve absolute quantification. Consequently, all changes in volatile abundance are expressed as normalized relative peak areas (semi-quantitative). One HS-GC-IMS analysis was performed per independent biological replicate (*n* = 3 per aging treatment).

### 2.7 Electronic tongue analysis

The filtrate of wine was analyzed for taste profiles using a SA402B Plus-EX electronic tongue (Intelligent Sensor Technology, Inc., Kanagawa, Japan) equipped with five bio-membrane-based sensors: AAE (umami), CT0 (saltiness), CA0 (sourness), C00 (bitterness), and AE1 (astringency). In addition to these five basic tastes, the system quantified bitterness and astringency aftertastes by measuring post-measurement potential differences. A reference solution simulating human oral cavity (30 mM KCl and 0.3 mM tartaric acid) was prepared for sensor calibration. All sensors were activated in this reference solution for 24 h prior to analysis. Taste values of wine samples were measured in triplicate against a reference electrode and normalized using the instrument's reference database.

### 2.8 Sensory evaluation using CATA

The sensory characteristics of unaged wine (control), wine aged for 12 months in oak barrels, and wine aged for 12 months in glass bottles were evaluated using the CATA method. A sensory lexicon was established via a focus group. Six experienced panelists (three females, three males; affiliated with Yantai University and excluded from subsequent CATA testing) independently evaluated the samples. By systematically characterizing sensory profiles and integrating Chinese vocabulary and context, preliminary descriptors for appearance, aroma, and taste attributes were identified through group discussion. The final lexicon included 10 appearance descriptors, 13 aroma descriptors, and 14 taste descriptors. To minimize position bias, the descriptor order was randomized using a Latin square design.

Sixty-one healthy consumers (42 females and 19 males aged from 19 to 22 years old) were recruited from the School of Life Sciences at Yantai University for CATA testing. All participants had no formal sensory training and reported no wine allergies. The sensory study protocols, including adherence to ethical guidelines, were approved by the School of Life Sciences of Yantai University. Prior to testing, subjects provided informed consent and received standardized training covering sensory evaluation procedures, the use of the 9-point hedonic scale (1 = ‘dislike extremely’, 9 = ‘like extremely’), and oral cleansing procedures (unsalted crackers followed by rinsing with room-temperature purified water).

Each wine sample (50 mL) was presented in a clear standard ISO wine glass covered to prevent the loss of volatiles. Samples were labeled with random three-digit codes and served in randomized order at room temperature. During each evaluation, participants sequentially: a) selected applicable appearance attributes and rated preference after visual inspection; b) selected applicable aroma attributes and rated preference after olfactory evaluation; c) selected applicable taste attributes and rated preference after tasting; d) rated overall preference. A mandatory 3-min rest interval with palate cleansers was enforced between samples to minimize fatigue. Finally, participants ranked their overall preference for all three wines.

Ethical statement: this sensory study, involving low-risk food preference evaluation with competent adult volunteers, was reviewed and approved by the School of Life Sciences, Yantai University. A formal ethical review by an institutional review board was not required under institutional policy for this study type. Informed consent was obtained from all participants prior to their voluntary involvement. Institutional guidelines were followed to safeguard participant rights, privacy, and well-being throughout the study.

### 2.9 Statistical analysis

All experiments and analyses were conducted in triplicate. Statistical analyses employed one-way ANOVA (*p* ≤ 0.05) and correspondence analysis in SPSS (version R27.0.1.0), while Duncan's multiple range test was applied for post-hoc comparisons of means. Cochran's Q test for the frequencies of sensory descriptors in CATA analysis was also conducted in SPSS. The mean and standard deviation were derived as the primary descriptive statistics using Excel 2021's AVERAGE and STDEVA functions. CIELab-derived color parameters (*L*^⁎^, *a*^⁎^, and *b*^⁎^) were visualized through R-generated color swatches (version 3.6.3). Other figures were produced using Origin (v9.8.0.200).

## 3 Results and discussion

### 3.1 Color properties and phenolic contents analysis

**Fig. 1:**
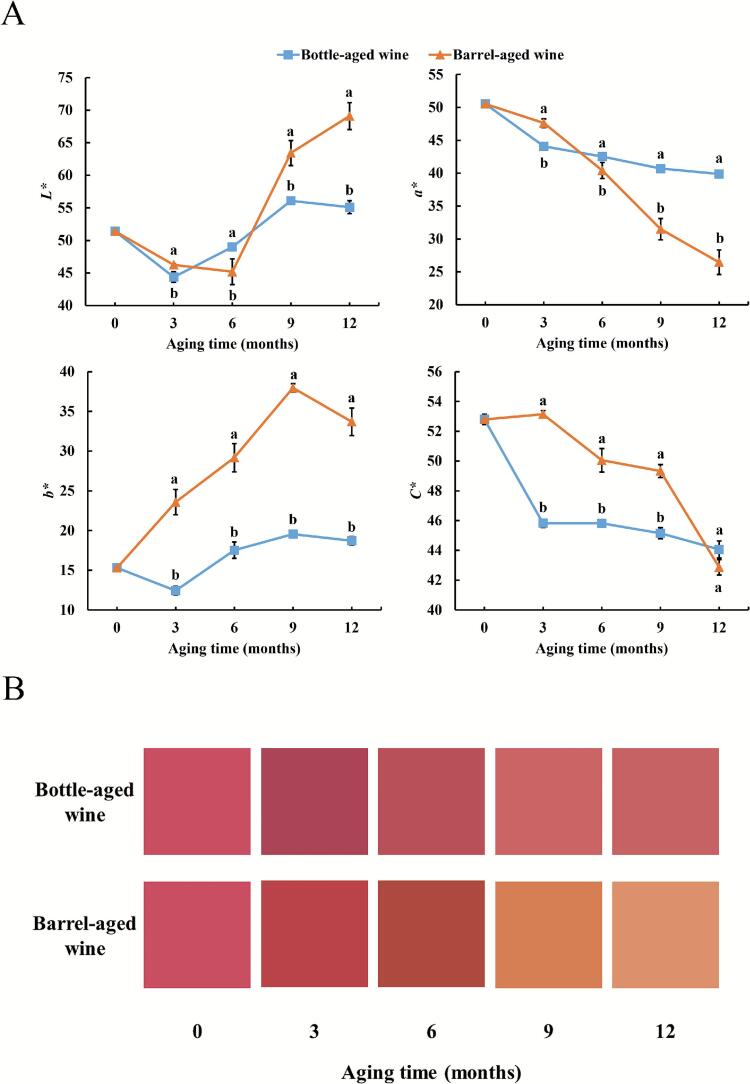
CIELab parameters of wines during aging (A). Color swatches of wines based on the CIE-*L*^⁎^*a*^⁎^*b*^⁎^ values (B).

As a fruit rich in anthocyanins, the color performance and stability of *Aronia melanocarpa* wines are critical sensory attributes for consumers. To precisely evaluate color variations throughout the aging process, the CIELab color space methodology was employed. As shown in Fig. 1A, *L*^*⁎*^ and *b*^⁎^values generally increased while *a*^⁎^ and *C*^⁎^ values decreased over time, indicating a progressive lightening and yellowing of all wines alongside reduced chromatic intensity (visually evident in Fig. 1B). After 12 months of aging, barrel-aged wine exhibited significantly higher *L*^*⁎*^ and *b*^⁎^ values, lower *a*^⁎^and *C*^⁎^ values, resulting in a distinct brownish-yellow hue compared to bottle-aged wine. This visually discernible difference (Δ*E*^*⁎*^ = 31.8) reflected divergent anthocyanin stability under the two aging conditions. While anthocyanin degradation was a major factor, the pronounced browning in barrel-aged wines was also consistent with the potential formation of polymeric pigments and tannin-anthocyanin adducts, processes influenced by higher oxygen ingress.

**Fig. 2:**
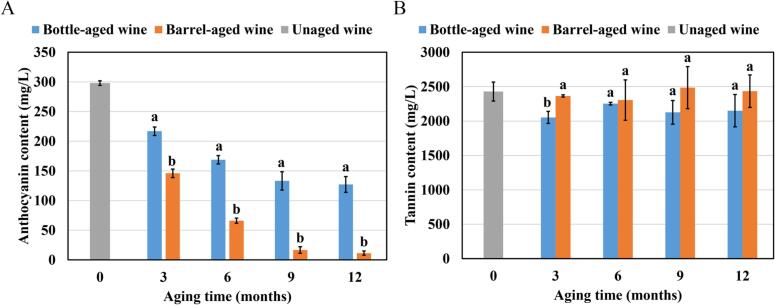
Contents of total anthocyanin (A) and tannin (B) of different wines.

The evolution of total anthocyanin content during aging is depicted in Fig. 2A. Total anthocyanins decreased significantly during aging, with bottle- and barrel-aged wines exhibiting 57.4% and 96.2% reductions, respectively, by the end of aging. Oak barrels act as active vessels due to their oxygen permeability, with the oxygen permeability estimated to range from 1 to 30 mg/L/year (Cui, Ling, Huang, Duan, & Lan, 2024). Although oxygen permeability was not directly measured in this study, the observed color shift and anthocyanin loss in barrel-aged wines were consistent with an enhanced oxidative environment compared to bottle aging. Previous studies identify cyanidin-3-*O*-galactoside, cyanidin-3-*O*-glucoside, cyanidin-3-*O*-arabinoside, and cyanidin-3-*O*-xyloside as the predominant pigments in *Aronia melanocarpa* berries (Dobros et al., 2024; Kubra Sasmaz, Kilic-Buyukkurt, Selli, Bouaziz, & Kelebek, 2024). It is well established that cyanidin-based anthocyanin glycosides, which contain a 1,2-dihydroxybenzene moiety, are highly susceptible to oxidation and degradation (Zhang et al., 2020). This explains why bottle-aged wine exhibited a more stable color compared to barrel-aged wine, as the restricted oxygen permeability of glass bottles decelerated anthocyanin degradation (Leleu et al., 2025).

Tannins are the key astringent compounds in *Aronia melanocarpa* juice (Huang et al., 2022; Tu et al., 2022), with a mean polymerization degree of 35.38. This value is notably higher than those found in wines produced from *Vitis vinifera* and hybrid grape varieties (Tu et al., 2022; Watrelot & Norton, 2020). As depicted in Fig. 2B, total tannin content remained relatively stable during aging, showing no pronounced change relative to the unaged wine. Therefore, the tannins in *Aronia melanocarpa* wine may have greater stability than those in red wines (Gambuti, Picariello, Rinaldi, Ugliano, & Moio, 2020; Tu et al., 2022).

### 3.2 Antioxidant capacity analysis

The antioxidant capacity of wine, primarily attributed to its phenolic compounds, is associated with potential health benefits (Dobros et al., 2024). To comprehensively evaluate changes in antioxidant capacity during aging, *Aronia melanocarpa* wines were analyzed using three established assays: DPPH, ABTS, and FRAP. These assays are widely utilized in the field of alcoholic beverages for their ability to provide a holistic assessment of antioxidant properties (Hao et al., 2025; Xu et al., 2025). Specifically, the FRAP assay measures the reducing capacity of the wine, which reflects its ability to donate electrons and neutralize oxidizing agents. Meanwhile, the ABTS and DPPH assays focus on evaluating the anti-radical activity, indicating the wine's capability to quench free radicals. Together, these assays provided a comprehensive understanding of the antioxidant profile of *Aronia melanocarpa* wines as they age.

**Fig. 3:**
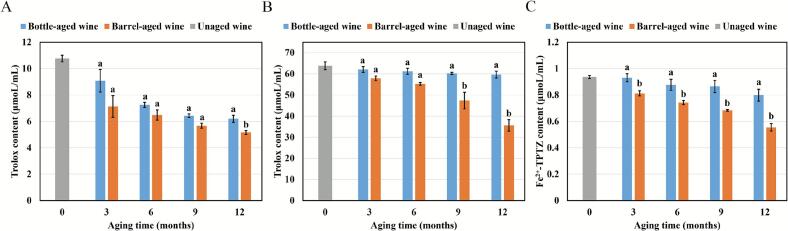
Antioxidant capacity of wines measured by DPPH (A), ABTS (B), and FRAP (C) assays.

The results of the antioxidant capacity tests are presented in Fig. 3. For all three methods employed, both bottle- and barrel-aged wines exhibited a significant decline in antioxidant capacity as the aging process progressed. This observation aligns with findings from previous studies (Wang, Wang, Tang, Rao, & Chen, 2022; Xie et al., 2024). However, contrasting results have been reported by other researchers, who found a significant increase in antioxidant capacity after 12 months of bottle storage (Alejandro Ruiz et al., 2024; Varo, Martín-Gómez, Merida, & Serratosa, 2023). This discrepancy may arise from the wide range of factors influencing antioxidant capacity, such as cultivar, growing conditions, and winemaking processes. Furthermore, the antioxidant activity of barrel-aged wine was consistently lower than that of bottle-aged wine. The more pronounced decline in barrel-aged wines aligned with their greater anthocyanin degradation (Fig. 2A) and suggested that barrel-induced micro‑oxygenation accelerated the evolution of antioxidant-active phenolics. This correlation reinforces the role of anthocyanins as key contributors to antioxidant activity and highlights how oxidative aging diminishes their redox potential.

### 3.3 Volatile compounds analysis by HS-GC-IMS

HS-GC-IMS was selected for this study because it aligns closely with the research objective: to compare the dynamic evolution of volatile profiles during bottle- versus barrel-aging. Unlike approaches focused on absolute quantification, HS-GC-IMS excels at tracking relative changes over time through visual, comparative analysis—making it particularly suitable for capturing subtle changes in complex aroma mixtures. The technique also offers practical advantages, including shorter analysis time, simpler sample preparation, and enhanced data visualization compared to methods such as HS-SPME-GC–MS (Zhu, Benkwitz, Sarmadi, & Kilmartin, 2021). It has been successfully applied in recent years to characterize the volatile profiles of various fruit wines (Zhang et al., 2025; Zhou et al., 2025). Therefore, HS-GC-IMS was employed to comprehensively monitor the volatile transformations occurring during the aging of *Aronia melanocarpa* wines.

**Fig. 4:**
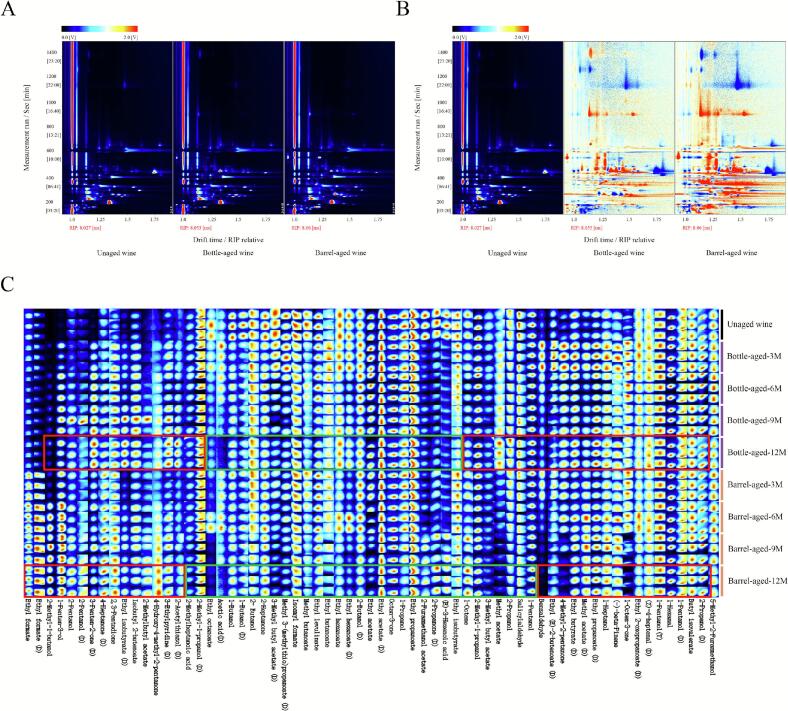
Volatile compounds analysis using HS-GC-IMS. The two-dimensional topographic plots (A) and the difference comparison topographic plots (B) for unaged wine, bottle- and barrel-aged wines after 12 months of aging. The dynamic fingerprints of wines during aging (C).

The topographic map of the volatile compounds is shown in Fig. 4A. The red line on the abscissa at 1.0 corresponds to the normalized reaction ion peak (RIP), with each dot positioned to its right representing a detected volatile compound. Most signals were observed within drift times of 8.0–16.0 ms and retention times of 200–600 s. Regions highlighted in red indicate higher concentrations of volatile compounds (expressed as peak area), while blue regions indicate lower concentrations. To better visualize the differences in volatile profiles during aging, difference comparison topographic plots were generated using the unaged wine as the reference (Fig. 4B). In these difference plots, a white background signifies compounds with concentrations similar to the reference, red indicates higher concentrations relative to the unaged wine; and blue indicates lower concentrations. The intensity of the color reflects the magnitude of the difference. Notably, the spectra of bottle- and barrel-aged wines exhibited distinct patterns of red and blue areas, indicating significant differences in volatile compound profiles after aging.

To intuitively illustrate the dynamic changes in volatile compounds throughout the aging of *Aronia melanocarpa* wines, a volatile compound fingerprint (Fig. 4C) was constructed using all peaks identified in the HS-GC-IMS two-dimensional spectra. A total of 65 volatile compounds were identified (detailed in Table S1), categorized into eight groups: 18 higher alcohols, 16 ethyl esters, 8 ketones, 6 acetate esters, 5 other esters, 4 acids, 3 aldehydes, 1 terpenoids, and 4 other compounds. Monomeric, dimeric (D), and trimeric (T) forms of compounds were detected at varying concentrations. This multiplicity of signals for individual compounds likely arises from the formation of adducts between monomeric ions and neutral molecules within the ion transfer region of the instrument (Zhai et al., 2024). These features were retained as separate analytical signals. Their peak areas were not summed for quantitative analysis due to the lack of authentic standards required to establish valid relative response factors across adducts.

As illustrated in Fig. 4C, volatile compounds within the red and green boxes exhibited relatively higher or lower levels, respectively, in wines after 12 months of aging compared to the unaged wine. For the bottle-aged wine, concentrations of several compounds increased markedly (over 10-fold), including 2-acetylthiazol (D), 2-methyl-1-butanol, 3-penten-2-one (D), 1-penten-3-ol, 4-hydroxy-4-methyl-2-pentanone, isobutyl-2-butenoate, ethyl isobutyrate (D), and 3-ethylpyridine (D). Among these, 2-acetylthiazol (D) contributed roasted nutty, meaty, and bready aromas (e.g., popcorn, stir-fried chestnuts, roasted oatmeal, roasted meat), 3-penten-2-one (D) imparted spicy notes, and 3-ethylpyridine (D) provided tobacco and leather nuances. Conversely, eight volatile compounds decreased substantially (over 70%), including methyl butanoate, acetic acid (D), (*E*)-3-hexenoic acid, 2-heptanone, ethyl isobutyrate, 2-propanone (D), 1-butanol, and ethyl butanoate. These diminished compounds are primarily associated with fresh and fruity aromas. Therefore, bottle aging promoted the development of tertiary aromas alongside a reduction in fresh fruity characteristics, enhancing the wine's aromatic complexity and maturity.

For the barrel-aged wine, significant increases (over 10-fold) were observed for 2-methyl-1-butanol, 2-acetylthiazol (D), 1-penten-3-ol, 3-penten-2-one (D), 4-hydroxy-4-methyl-2-pentanone, isobutyl-2-butenoate, ethyl isobutyrate (D), 4-heptanone (D), and 2-pentanol (D). 2-Acetylthiazol (D) again contributed roasted aromas as described above. Notably, increased levels of 2-methyl-1-butanol and 4-heptanone (D) were associated with cocoa and coconut aromas. Furthermore, elevated 3-penten-2-one and 4-hydroxy-4-methyl-2-pentanone, while possessing fruity aspects, are recognized key markers of wine oxidation (Bueno et al., 2018; Pinto et al., 2018). This suggests that oxidative reactions may have been more pronounced in barrels. A more pronounced reduction occurred in barrel-aged wine, with twenty-one compounds decreasing by over 70%, including 2-methyl-1-propanol, methyl acetate, ethyl octanoate, ethyl acetate, 4-methyl-2-pentanone, benzaldehyde, ethyl levulinate, 1-propanol, salicylaldehyde, 2-butanol, 3-methyl-butylacetate, 2-butanol (D), ethyl butanoate, methyl butanoate, ethyl hexanoate (D), 1-octen-3-one, 3-methyl-butylacetate (D), isoamyl formate, ethyl acetate (D), 1-butanol, and 2-propanol. Compared to bottle aging, barrel aging induced a significantly greater decline in volatile compounds responsible for fresh and fruity notes. The substantial loss of fresh and fruity volatiles may not only result from direct oxidation but could also be influenced by chemical interactions with oak wood extractives (e.g., ellagitannins).

**Fig. 5:**
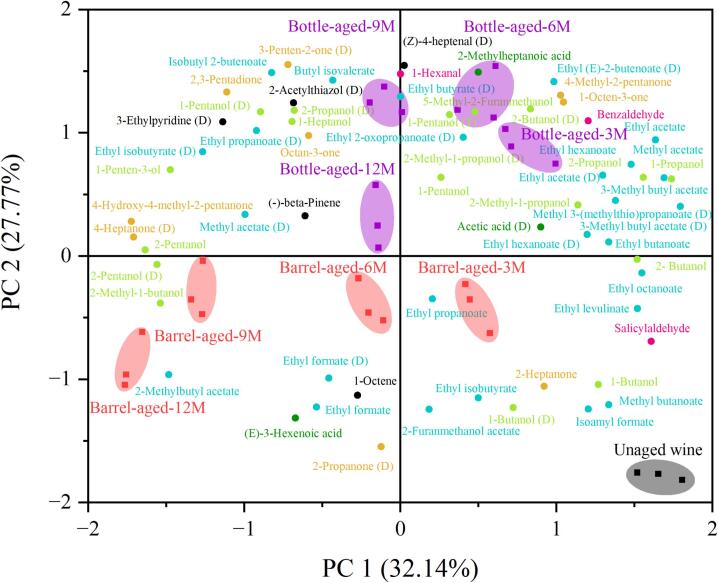
Principal component analysis (PCA) of volatile compounds.

Principal component analysis (PCA) was applied to the multiple variables obtained from HS-GC-IMS to differentiate volatile compound profiles. As shown in Fig. 5, the first two principal components (PCs) collectively accounted for 59.4% of the total variance. Although this value is not exceptionally high, it is acceptable in PCA of complex volatile datasets, where numerous minor compounds contribute to total variance. The close proximity of data points from replicate experiments indicated high experimental reproducibility. In contrast, the distinct separation between data points representing bottle- and barrel-aged wines reflected significant differences in their aroma composition. These multivariate statistical results further validated the significant impact of aging vessels and time on the aroma profile of *Aronia melanocarpa* wines.

### 3.4 Taste analysis by electronic tongue

**Fig. 6:**
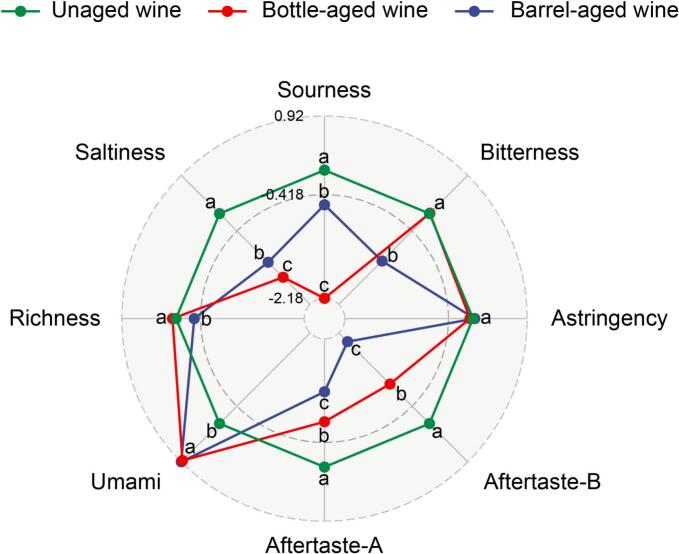
The taste profiles measured by electronic tongue of unaged wine, bottle- and barrel-aged wines after 12 months of aging. Data are expressed as differences from the unaged wine.

An electronic tongue was employed to characterize the overall taste profiles of *Aronia melanocarpa* wines before aging and after 12 months of aging. This technology is user-friendly, rapid, and objective, and has been widely utilized in the analysis of alcoholic beverages, including Baijiu (Chinese liquor), beer, and wine (Garcia-Hernandez, Garcia-Cabezon, Rodriguez-Mendez, & Martin-Pedrosa, 2025; Lu, Hu, Hu, Li, & Tian, 2022). To facilitate comparison, taste response values were normalized relative to the unaged wine (assigned a value of zero), as illustrated in Fig. 6. Both aging methods significantly reduced the intensities of saltiness, sourness, aftertaste-A (astringency aftertaste), and aftertaste-B (bitter aftertaste). Notably, bottle aging exhibited a greater reduction effect on saltiness and sourness compared to barrel aging, whereas barrel aging resulted in a more pronounced decrease in astringency/bitter aftertastes. Umami intensity was enhanced by both aging methods to a similar extent. It should be noted that the electronic tongue's umami sensor (AAE) is designed to respond to certain glutamates and nucleotides, but may also cross-react with peptides or organic acids present in wine. This may explain why the instrument detected increased umami signal, whereas human perception of umami in wine is generally masked by other dominant taste and aroma components. Electronic tongue outputs thus reflect the presence of taste-active compounds rather than direct human taste perception.

Regarding richness and bitterness, bottle aging showed no significant impact; however, barrel aging significantly reduced both attributes. Therefore, the aging process has been shown to improve the taste profiles of *Aronia melanocarpa* wines to some extent by reducing undesirable sensory characteristics. Furthermore, astringency intensity remained unaffected by either aging method, consistent with the stable total tannin content.

### 3.5 Sensory analysis by CATA

**Fig. 7:**
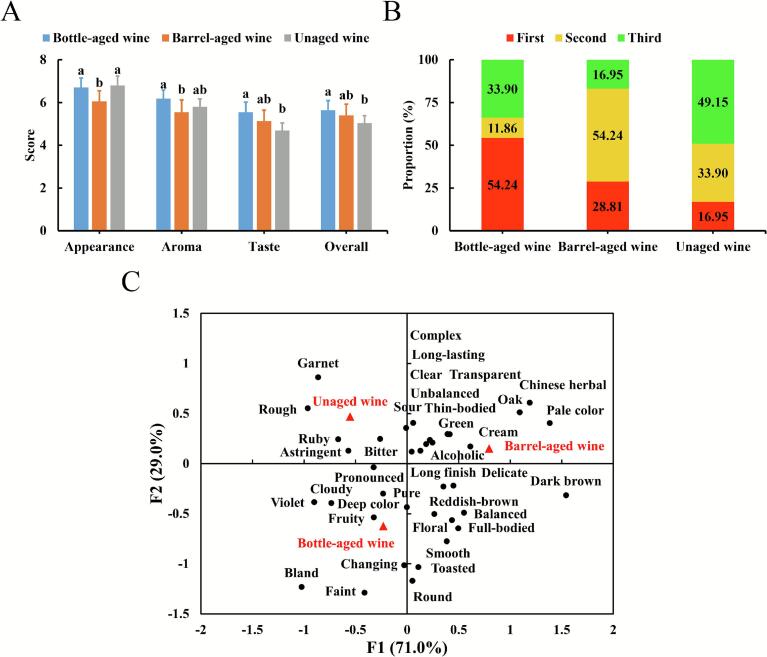
Sensory evaluation using CATA. Participants' mean hedonic scores (A), ranking (B), and correspondence analysis of the sensory descriptors (C) for unaged wine, bottle- and barrel-aged wines after 12 months of aging.

CATA is a simple, rapid and reliable method for gathering consumer perception data on the sensory characteristics of diverse food products (Antille et al., 2024; Li & He, 2023). Complementing the CATA task, consumer preference was assessed using a 9-point hedonic scale for the appearance, aroma, taste, and overall quality of the wines (Fig. 7A). Regarding appearance, the score for barrel-aged wine was significantly lower than those for unaged and bottle-aged wines (*p* ≤ 0.05). This difference may be attributed to the development of a brownish-yellow hue in the barrel-aged wine, resulting from anthocyanin degradation and oxidation. For aroma, barrel-aged wine received a significantly lower score (*p* ≤ 0.05) than bottle-aged wine. No significant differences were observed between the unaged wine and either of the aged wines, suggesting a potential consumer preference against the specific aroma profile developed during barrel aging. In taste evaluation, bottle-aged wine achieved significantly higher preference scores (*p* ≤ 0.05). This finding aligns with the multidimensional taste response profile obtained via electronic tongue analysis, further supporting the optimizing effect of bottle aging on the wine's taste quality. Concerning overall preference, bottle-aged wine scored significantly higher than unaged wine (*p* ≤ 0.05), while no significant difference was observed between barrel-aged and unaged wines.

To gain deeper insights into consumer preference, participants were also required to rank the three wines (Fig. 7B). Bottle-aged wine was ranked first by 54.24% of consumers, a proportion significantly higher (*p* ≤ 0.05) than that for the other wines. Barrel-aged wine ranked second overall, receiving first-place preference from 28.81% of participants, while unaged wine had a first-preference rate below 20%. Collectively, the analysis of both hedonic scores and ranking data demonstrated that both aging methods significantly enhance the sensory characteristics of *Aronia melanocarpa* wine. Notably, bottle aging exhibited a more pronounced advantage, particularly in terms of appearance and taste.

Table 1 summarizes the frequencies of sensory descriptors selected by participants in the CATA analysis. For the appearance descriptors, ‘transparent’ (75), ‘deep color’ (63), ‘clear’ (54), ‘reddish-brown’ (51), ‘ruby’ (42), and ‘garnet’ (41) were most frequently selected. Significant differences (indicated by asterisks) were observed among descriptors related to color hue (i.e., ‘violet’, ‘ruby’, ‘garnet’, ‘reddish-brown’, and ‘dark brown’), confirming that consumers could visually distinguish the color evolution induced by aging. Regarding aroma descriptors, ‘pronounced’ (74), ‘fruity’ (64), ‘Chinese herbal’ (52), and ‘complex’ (41) exhibited the highest selection frequencies. Notably, the frequency of ‘fruity’ was lower in the barrel-aged wine (14) compared to the unaged wine (20), while the bottle-aged wine showed the highest frequency (30). In contrast, ‘Chinese herbal’ and ‘oak’ aromas were more frequently associated with barrel-aged wine (35 and 18 selections, respectively), underscoring the impact of wood contact on aromatic profile. For taste descriptors, ‘astringent’ (123), ‘sour’ (81), ‘bitter’ (73), ‘rough’ (48), and ‘alcoholic’ (46) were the most frequently selected attributes, confirming their central role in defining the palate of *Aronia melanocarpa* wines. Crucially, the frequencies of ‘astringent’, ‘bitter’, and ‘rough’ were markedly reduced in the aged wines, particularly in the barrel-aged wine (23, 20, and 5, respectively, vs. unaged wine: 57, 31, 29). This reduction aligned with electronic tongue results, which indicated the barrel-aged wine had the lowest intensities for bitterness and astringency/bitterness aftertastes. Collectively, these findings suggested that both bottle and barrel aging, especially the latter, can mitigate excessive astringency, bitterness and roughness, thereby improving the taste quality of *Aronia melanocarpa* wine.

**Table 1:** Frequencies of sensory descriptors selected by participants in CATA analysis.

Sensory characteristics	Descriptors	Unaged wine	Bottle-aged wine	Barrel-aged wine	Sum
Appearance	Violet***	12	13	2	27
	Ruby*	21	14	7	42
	Garnet***	26	9	6	41
	Reddish-brown*	8	20	23	51
	Dark brown***	0	7	18	25
	Deep color	21	26	16	63
	Pale color***	2	2	10	14
	Transparent**	23	18	34	75
	Clear	18	16	20	54
	Cloudy	7	8	2	17
Aroma	Fruity**	20	30	14	64
	Floral	5	10	9	24
	Green	12	10	15	37
	Chinese herbal***	11	6	35	52
	Oak**	6	4	18	28
	Cream	2	2	4	8
	Toasted	2	7	4	13
	Pure	7	11	8	26
	Complex	16	10	15	41
	Long-lasting	9	7	13	29
	Pronounced	29	27	18	74
	Faint**	4	12	3	19
	Changing	2	6	3	11
Taste	Sour	32	21	28	81
	Bitter*	31	22	20	73
	Astringent***	57	43	23	123
	Alcoholic	16	14	16	46
	Round	2	8	4	14
	Smooth	4	13	11	28
	Full-bodied	3	9	9	21
	Thin-bodied	6	5	7	18
	Bland*	3	6	0	9
	Delicate	7	11	14	32
	Rough***	29	14	5	48
	Balanced	2	5	5	12
	Unbalanced	5	4	6	15
	Long finish	8	12	14	34

Asterisks indicate significant difference according to Cochran's Q test at * *p* ≤ 0.05, ** *p* ≤ 0.01, and *** *p* ≤ 0.001, respectively.

Correspondence analysis (CA) was performed to generate a sensory map visualizing the associations between the wine samples and sensory attributes (Fig. 7C). The first two dimensions, which are presented in the map, captured the major structure of the data, explaining 70.9% and 29.1% of the total inertia. Together, they accounted for the entirety of the inertia represented in this two-dimensional solution. The three wine samples occupied distinct quadrants within the map, suggesting perceivable sensory differences among untrained consumers. Unaged wine was positioned in the second quadrant and exhibited positive correlations with the descriptors ‘ruby’, ‘rough’, ‘bitter’, ‘sour’, and ‘astringent’. Bottle-aged wine was located in the third quadrant and showed associations with ‘deep color’, ‘fruity’, and ‘pure’ attributes. Barrel-aged wine was situated in the first quadrant and demonstrated positive correlations with ‘pale color’, ‘Chinese herbal’, ‘oak’, and ‘cream’ attributes, evidencing the dual impact of oxidative and extractive phenomena during barrel maturation.

A limitation of this sensory study should be noted. The consumer panel comprised university students aged 19–22 from a single institution. This homogeneous sample facilitated controlled discrimination of the sensory attributes under investigation. Nevertheless, the results may not be fully generalizable to a broader consumer population with more diverse age, cultural backgrounds, and wine consumption experience. Therefore, future studies involving a larger and more demographically varied panel are warranted to confirm the broader market applicability of these findings.

It is important to consider the scale of the aging vessels used in this study. The 8-L new oak barrels possess a significantly higher surface-area-to-volume ratio compared to the 225-L barrels standard in commercial winemaking. This difference is known to accelerate both oxygen permeation and the extraction of wood-derived compounds. Consequently, the chemical and sensory changes observed in our barrel-aged wines, such as the pronounced color browning, rapid anthocyanin degradation, and development of oak-related aromas, may represent an intensified version of the aging process that would occur over a longer period in larger barrels. While this model system validly demonstrates the comparative directionality and nature of changes induced by oak contact relative to bottle aging, direct extrapolation of the observed reaction kinetics to industrial-scale practice should be undertaken with caution.

## 4 Conclusion

This study provided the first systematic evidence that aging vessels profoundly influence the chemical and sensory properties of *Aronia melanocarpa* wines. Oak barrels accelerated anthocyanin degradation (96.2% loss), leading to a reddish-brown hue and higher levels of oxidative aroma markers (e.g., 3-penten-2-one and 4-hydroxy-4-methyl-2-pentanone) and oak-derived notes (e.g., cocoa and coconut), but compromised antioxidant capacity. Glass bottles better preserved anthocyanins (42.6% retained), color intensity, and fresh fruity aromas while developing complex tertiary notes (e.g., spicy, tobacco, and leather). Both aging methods reduced sourness, aftertastes of astringency and bitterness (validated by electronic tongue), with barrel aging showing stronger mitigation of aftertastes. Critically, consumer testing (CATA) revealed a clear preference for bottle-aged wines (54.24% first-choice ranking) due to superior appearance, fruity aroma, and balanced taste, whereas barrel-induced browning, oak notes, and reduced fruity aroma lowered hedonic scores. The findings provide targeted guidance for selecting aging protocols that balance color stability, flavour complexity, and market acceptance in *Aronia melanocarpa* winemaking.

## CRediT authorship contribution statement

**Zhongzheng Zhang:** Writing – original draft, Methodology, Investigation, Formal analysis. **Qichen Yuan:** Writing – review & editing, Methodology, Formal analysis. **Hailong Xu:** Resources, Investigation, Conceptualization. **Mingbo Li:** Supervision, Methodology, Investigation. **Xu Zhao:** Writing – review & editing, Supervision, Resources, Methodology, Funding acquisition, Conceptualization.

## Declaration of competing interest

The authors declare that they have no known competing financial interests or personal relationships that could have appeared to influence the work reported in this paper.

## Data Availability

Data will be made available on request.

## Appendix A Supplementary data

Supplementary material Image 1
