# Supplementary material for: From chemical signatures to consumer preference: Decoding bottle and barrel aging effects on Aronia melanocarpa wines using integrated analytical and sensory techniques
Source: Food Chem X. 2026 Jan 13;33:103537. doi: 10.1016/j.fochx.2026.103537 (PMC12830279; doi:10.1016/j.fochx.2026.103537)
Supplement: Supplementary file 1 — Supplementary material [file mmc1.docx]

**Table S1.** Information of volatile components identified by HS-GC-IMS

| Components | Descriptors | CAS | Formula | Molecular weight | Retention index | Retention time (second) | Drift time (a.u.) |
| --- | --- | --- | --- | --- | --- | --- | --- |
| **Higher alcohols** |  |  |  |  |  |  |  |
| 1-Propanol | Alcohol, pungent | C71238 | C_3_H_8_O | 60.1 | 1074.3 | 383.965 | 1.11215 |
| 2-Propanol | Alcohol, spicy | C67630 | C_3_H_8_O | 60.1 | 934.2 | 254.003 | 1.09725 |
| 2-Propanol (D) | Alcohol, spicy | C67630 | C_3_H_8_O | 60.1 | 939.4 | 257.698 | 1.21127 |
| 1-Butanol | Wine | C71363 | C_4_H_10_O | 74.1 | 1160.8 | 524.832 | 1.18131 |
| 1-Butanol (D) | Wine | C71363 | C_4_H_10_O | 74.1 | 1160.8 | 524.832 | 1.37523 |
| 2-Butanol | Fruity | C78922 | C_4_H_10_O | 74.1 | 1016.3 | 321.692 | 1.13383 |
| 2-Butanol (D) | Fruity | C78922 | C_4_H_10_O | 74.1 | 1031.3 | 336.788 | 1.3402 |
| 2-Methyl-1-propanol | Fresh, alcoholic, leather | C78831 | C_4_H_10_O | 74.1 | 1109.4 | 431.278 | 1.17171 |
| 2-Methyl-1-propanol (D) | Fresh, alcoholic, leather | C78831 | C_4_H_10_O | 74.1 | 1110 | 432.281 | 1.36077 |
| 1-Penten-3-ol | Ethereal, green, tropical fruity | C616251 | C_5_H_10_O | 86.1 | 1194.6 | 595.513 | 0.94146 |
| 1-Pentanol | Balsamic | C71410 | C_5_H_12_O | 88.1 | 1221.4 | 641.718 | 1.24393 |
| 1-Pentanol (D) | Balsamic | C71410 | C_5_H_12_O | 88.1 | 1222.1 | 643.104 | 1.49037 |
| 1-Pentanol (T) | Balsamic | C71410 | C_5_H_12_O | 88.1 | 1227.5 | 652.806 | 1.79337 |
| 2-Pentanol | Fusel oil, Green | C6032297 | C_5_H_12_O | 88.1 | 1134.4 | 474.474 | 1.21161 |
| 2-Pentanol (D) | Fusel oil, Green | C6032297 | C_5_H_12_O | 88.1 | 1136.7 | 478.632 | 1.44593 |
| 2-Methyl-1-butanol | Roast onion, fruity, floral, wine | C137326 | C_5_H_12_O | 88.1 | 1193.5 | 593.67 | 1.22373 |
| 5-Methyl-2-Furanmethanol | Sweet caramel | C3857258 | C_6_H_8_O_2_ | 112.1 | 970.8 | 281.719 | 1.26116 |
| 1-Heptanol | Grape, fruity, wine, violet, peony | C111706 | C_7_H_16_O | 116.2 | 971.9 | 282.598 | 1.39721 |
| **Aldehydes** |  |  |  |  |  |  |  |
| 1-Hexanal | Fresh, green, fat, fruity | C66251 | C_6_H_12_O | 100.2 | 1109.2 | 430.943 | 1.24505 |
| Benzaldehyde | Bitter almond, cherry, nutty | C100527 | C_7_H_6_O | 106.1 | 1529.9 | 1420.086 | 1.15184 |
| Salicylaldehyde | Medical, spicy, cinnamon wintergreen cooling | C90028 | C_7_H_6_O_2_ | 122.1 | 1038.9 | 344.728 | 1.13179 |
| **Ketones** |  |  |  |  |  |  |  |
| 2-Propanone (D) | Fresh, apple, pear | C67641 | C_3_H_6_O | 58.1 | 811.9 | 193.547 | 1.11055 |
| 3-Penten-2-one (D) | Fruity, turns into spicy during storage | C625332 | C_5_H_8_O | 84.1 | 1141.7 | 487.872 | 1.33281 |
| 2,3-Pentadione | Sweet, cream, caramel, nuts, cheese | C600146 | C_5_H_8_O_2_ | 100.1 | 1042.9 | 348.901 | 1.22488 |
| 4-Methyl-2-pentanone | Ketone | C108101 | C_6_H_12_O | 100.2 | 992 | 299.174 | 1.48014 |
| 2-Heptanone | Pear, banana, fruity, slight medicinal fragrance | C110430 | C_7_H_14_O | 114.2 | 1161.3 | 525.756 | 1.25605 |
| 4-Heptanone (D) | Fruity | C123193 | C_7_H_14_O | 114.2 | 1135.6 | 476.579 | 1.5885 |
| 4-Hydroxy-4-methyl-2-pentanone | Mild, pleasant | C123422 | C_6_H_12_O_2_ | 116.2 | 1366.2 | 941.918 | 1.13963 |
| 1-Octen-3-one | Strong earthy, mushroom, vegetable, fishy, chicken | C4312996 | C_8_H_14_O | 126.2 | 992.2 | 299.314 | 1.27406 |
| Octan-3-one | Mouldy, ketone, green, waxy, vegetable, mushroom, cheese, fruity | C106683 | C_8_H_16_O | 128.2 | 1220.1 | 639.408 | 1.31261 |
| **Ethyl esters** |  |  |  |  |  |  |  |
| Ethyl formate | Spicy and stimulating taste, pineapple, rum | C109944 | C_3_H_6_O_2_ | 74.1 | 826.7 | 199.616 | 1.06487 |
| Ethyl formate (D) | Spicy and stimulating taste, pineapple, rum | C109944 | C_3_H_6_O_2_ | 74.1 | 828.9 | 200.527 | 1.21693 |
| Ethyl acetate | Fresh, fruity, sweet, grassy | C141786 | C_4_H_8_O_2_ | 88.1 | 881.8 | 223.946 | 1.0966 |
| Ethyl acetate (D) | Fresh, fruity, sweet, grassy | C141786 | C_4_H_8_O_2_ | 88.1 | 893.9 | 229.683 | 1.33761 |
| Ethyl propanoate | Grape, pineapple, fruity, rum | C105373 | C_5_H_10_O_2_ | 102.1 | 949.7 | 265.385 | 1.15491 |
| Ethyl propanoate (D) | Grape, pineapple, fruity, rum | C105373 | C_5_H_10_O_2_ | 102.1 | 971 | 281.859 | 1.4531 |
| Ethyl 2-oxopropanoate (D) | Fruity, sweet rum, vegetable caramel | C617356 | C_5_H_8_O_3_ | 116.1 | 1250.6 | 696.234 | 1.44593 |
| Ethyl (E)-2-butenoate (D) | Sourness, fruity, rum ether | C623701 | C_6_H_10_O_2_ | 114.1 | 1137.6 | 480.187 | 1.54374 |
| Ethyl isobutyrate | Sweet, fruity, alcoholic, rummy | C97621 | C_6_H_12_O_2_ | 116.2 | 981.4 | 290.248 | 1.20674 |
| Ethyl isobutyrate (D) | Sweet, fruity, alcoholic, rummy | C97621 | C_6_H_12_O_2_ | 116.2 | 979.8 | 288.973 | 1.56048 |
| Ethyl butyrate (D) | Pineapple, fruity, ester, whiskey | C105544 | C_6_H_12_O_2_ | 116.2 | 1052.9 | 359.739 | 1.55918 |
| Ethyl butanoate | Pineapple, fruity, ester, whiskey | C105544 | C_6_H_12_O_2_ | 116.2 | 1009.5 | 315.112 | 1.20414 |
| Ethyl levulinate | Apple | C539888 | C_7_H_12_O_3_ | 144.2 | 1070.5 | 379.502 | 1.19378 |
| Ethyl hexanoate | Pineapple, fruity, wine | C123660 | C_8_H_16_O_2_ | 144.2 | 1250.4 | 695.873 | 1.33918 |
| Ethyl hexanoate (D) | Pineapple, fruity, wine | C123660 | C_8_H_16_O_2_ | 144.2 | 1249.6 | 694.386 | 1.79943 |
| Ethyl octanoate | Fruity, pineapple, apple, brandy | C106321 | C_10_H_20_O_2_ | 172.3 | 1453.6 | 1172.728 | 1.47523 |
| **Acetate esters** |  |  |  |  |  |  |  |
| Methyl acetate | Ethereal | C79209 | C_3_H_6_O_2_ | 74.1 | 834.5 | 202.907 | 1.03181 |
| Methyl acetate (D) | Ethereal | C79209 | C_3_H_6_O_2_ | 74.1 | 830.3 | 201.13 | 1.19258 |
| 2-Furanmethanol acetate | Sweet, banana | C623176 | C_7_H_8_O_3_ | 140.1 | 994.3 | 301.086 | 1.42183 |
| 2-Methylbutyl acetate | Fruity | C624419 | C_7_H_14_O_2_ | 130.2 | 1133.5 | 472.795 | 1.28775 |
| 3-Methyl butyl acetate | Sweet, banana, fruity | C123922 | C_7_H_14_O_2_ | 130.2 | 1132.6 | 471.24 | 1.30453 |
| 3-Methyl butyl acetate (D) | Sweet, banana, fruity | C123922 | C_7_H_14_O_2_ | 130.2 | 1135.4 | 476.322 | 1.75297 |
| **Other esters** |  |  |  |  |  |  |  |
| Methyl butanoate | Apple, sweet banana, pineapple | C623427 | C_5_H_10_O_2_ | 102.1 | 979.8 | 288.956 | 1.141 |
| Methyl 3-(methylthio)propanoate (D) | Pineapple | C13532188 | C_5_H_10_O_2_S | 134.2 | 1028.7 | 334.121 | 1.61241 |
| Isoamyl formate | Plum, blackcurrant | C110452 | C_6_H_12_O_2_ | 116.2 | 1062.1 | 369.939 | 1.27023 |
| Isobutyl 2-butenoate | Fruity, jam | C589662 | C_8_H_14_O_2_ | 142.2 | 979.4 | 288.688 | 1.31365 |
| Butyl isovalerate | Bananas, blue cheese | C109193 | C_9_H_18_O_2_ | 158.2 | 1058.1 | 365.476 | 1.38814 |
| **Acids** |  |  |  |  |  |  |  |
| Acetic acid (D) | Spicy | C64197 | C_2_H_4_O_2_ | 60.1 | 1494.8 | 1300.556 | 1.05705 |
| (E)-3-Hexenoic acid | Fruity, fresh | C1577180 | C_6_H_10_O_2_ | 114.1 | 998.7 | 304.961 | 1.2263 |
| 2-Methylheptanoic acid | Butter | C1188029 | C_8_H_16_O_2_ | 144.2 | 1132.1 | 470.264 | 1.40658 |
| **Terpenoid** |  |  |  |  |  |  |  |
| (-)-beta-Pinene | Dry woody, herbal | C18172673 | C_10_H_16_ | 136.2 | 963.8 | 276.196 | 1.21087 |
| **Others** |  |  |  |  |  |  |  |
| 1-Octene | Gasoline | C111660 | C_8_H_16_ | 112.2 | 821.7 | 197.525 | 1.15279 |
| (Z)-4-Heptenal (D) | Grass, oil | C6728310 | C_7_H_12_O | 112.2 | 1247.9 | 691.152 | 1.63379 |
| 3-Ethylpyridine (D) | Tobacco, leather | C536787 | C_7_H_9_N | 107.2 | 976.9 | 286.647 | 1.50631 |
| 2-Acetylthiazol (D) | Popcorn, stir fried chestnuts, roasted oatmeal, roasted meat, nutty, bread | C24295032 | C_5_H_5_NOS | 127.2 | 1028.7 | 334.145 | 1.47478 |
